# Nonlinear Analytics for Electrochemical Biosensor Design Using Enzyme Aggregates and Delayed Mass Action

**DOI:** 10.3390/s22030980

**Published:** 2022-01-27

**Authors:** Vasyl Martsenyuk, Aleksandra Klos-Witkowska, Sergei Dzyadevych, Andriy Sverstiuk

**Affiliations:** 1 Department of Computer Science and Automation, University of Bielsko-Biala, 43-309 Bielsko-Biala, Poland; awitkowska@ath.bielsko.pl; 2 Institute of Molecular Biology and Genetics, National Academy of Sciences of Ukraine, 150 Zabolotnogo St., 03143 Kiev, Ukraine; dzyad@yahoo.com; 3 Medical Informatics Department, Ternopil National Medical University, 46001 Ternopil, Ukraine; sverstyuk@tdmu.edu.ua

**Keywords:** electrochemical biosensor, enzyme kinetics, Michaelis–Menten model, Brown’s model, mass action law, substrate, inhibitor, time delays, parameter identification

## Abstract

The paper is devoted to the extension of Brown’s model of enzyme kinetics to the case with distributed delays. Firstly, we construct a multi-substrate multi-inhibitor model using discrete and distributed delays. Furthermore, we consider simplified models including one substrate and one inhibitor, for which an experimental study has been performed. The algorithm of parameter identifications was developed which was tested on the experimental data of solution conductivity. Both the model and Kohlrausch’s law parameters are obtained as a result of the optimization procedure. Comparison of plots constructed with the help of the estimated parameters has shown that in such case the model with distributed delays is more chemically adequate in comparison with the discrete one. The methods of generalization of the results to the multi-substrate multi-inhibitor cases are discussed.

## 1. Introduction

Delayed systems play an important role in chemical kinetics [1,2]. Even for the complex network of the first order reactions, it can be described by relatively simple system of delayed differential equations, in which the effects of intermediates are replaced by time lags [3,4].

The interaction between two chemicals A and B, forming the product C, is not instant but is during some time interval τ>0. Hence, the law of mass action as the fundamental law of chemical kinetics should be reformulated schematically as:

$$\begin{array}{c} A\left(t\right)+B\left(t\right)\overset{k}{⟶}C(t+\tau ) \end{array}$$

leading us to the differential equation with delay:

(1) $$\frac{dC(t)}{dt}=kA\left(t-\tau \right)B\left(t-\tau \right),$$

which is called the law of delayed mass action.

Since delay τ is rather a random variable than a deterministic one and can accept various values according to some distribution laws, here, we offer a considerably more advanced model which includes continuously distributed delays (or simply, distributed delays), which can be described by the differential equation with distributed delay:

(2) $$\frac{dC(t)}{dt}=k\int_{-\infty }^{0}f(s)A(t+s)B(t+s)ds,$$

where f(s) is the probability density function of time delay τ. The model (2) can be referred to as the law of mass action with distributed delay. In practice, we can consider an integral with limited bounds in (2), using the properties of the distribution of random variables such as Chebyshev’s inequality, as it will be shown in Section 3.1.

The reactions which are used in electrochemical biosensing come from the reactions that are catalyzed by an enzyme. They are commonly known as reversible [5] or irreversible [6] reactions. The irreversible one-complex Michaelis–Menthen (IR1CMM) mechanism is a keystone in modeling enzyme kinetics. Its reaction scheme

$$\begin{array}{c} E+S\overset{k1}{\underset{k_{-1}}{⇌}}C\overset{k2}{⟶}E+P \end{array}$$

represents a two-step process [7,8,9], where the enzyme E combines with the substrate S to form a complex C, which then breaks down into the product P, releasing E in the process. The mechanism IR1CMM is described with the help of ordinary differential equations:

(3) $$\begin{array}{cc} \frac{dn_{S}}{dt} & =k_{-1}n_{C}-k_{1}n_{S}n_{E},\\ \frac{dn_{E}}{dt} & =\left(k_{-1}+k_{2}\right)n_{C}-k_{1}n_{S}n_{E},\\ \frac{dn_{C}}{dt} & =k_{1}n_{S}n_{E}-\left(k_{2}+k_{-1}\right)n_{C},\\ \frac{dn_{P}}{dt} & =k_{2}n_{C}. \end{array}$$

Here, for any substance A, we denote its concentration at instant *t* as $n_{A}\left(t\right)$.

An application of the delayed mass action law to enzyme kinetics was inspired by Brown’s model, formulated in [10], where complex C has a lifetime τ before being decayed. We call the following reaction scheme:

$$\begin{array}{c} E\left(t\right)+S\left(t\right)\overset{kd}{⟶}E(t+\tau )+P(t+\tau ) \end{array}$$

the irreversible one-complex Brown’s (IR1CB) mechanism, which can be described with the following system of delayed differential equations:

(4) $$\begin{array}{cc} \frac{dn_{S}\left(t\right)}{dt} & =-k_{d}n_{E}\left(t\right)n_{S}\left(t\right),\\ \frac{dn_{E}\left(t\right)}{dt} & =-k_{d}n_{E}\left(t\right)n_{S}\left(t\right)+k_{d}n_{E}\left(t-\tau \right)n_{S}\left(t-\tau \right),\\ \frac{dn_{P}\left(t\right)}{dt} & =k_{d}n_{E}\left(t-\tau \right)n_{S}\left(t-\tau \right). \end{array}$$

When comparing mechanisms IR1CMM and IR1CB, Roussel, M.R has introduced the notion of a chemically acceptable model [1]. While evidencing that the model using delayed mass action law is more adequate, the most significant failure of the lag model was that the solutions of these equations oscillate around the equilibrium point, which is forbidden by the law of microscopic reversibility. Oscillatory enzyme reactions are found in a number of enzymatic systems. Goldbeter, A. investigated the influence of Michaelis–Menten kinetics on the oscillatory behavior in an enzyme system [11].

Albornoz, J.M. and Parravano, A. have also shown that the models based on delayed differential equations (DDE) oscillate at small values of the Michaelis–Menthen constant, which cannot be seen with the help of ordinary differential equations (ODE) [12]. Piephoff, D.E. et al. has described the conformation of non-equilibrium enzyme kinetics, where a traditional Michaelis–Menten model is extended to a generalized form, which includes corrections coming from informational currents within combined cyclic kinetics loops [13].

Hinch, R. and Schnell, S. studied the conditions of equivalence of enzyme–substrate reaction mechanisms involving multiple complexes with a distributed delay system without complexes [14]. It was shown that the distribution of the delay is determined by the number of intermediate complexes and the rates of the individual reaction mechanisms.

In continuing the research [14] and applying the mass action law with the distributed delay (2), we offer the following continuously distributed delay model:

(5) $$\begin{array}{cc} \frac{dn_{S}\left(t\right)}{dt} & =-k_{d}n_{E}\left(t\right)n_{S}\left(t\right),\\ \frac{dn_{E}\left(t\right)}{dt} & =-k_{d}n_{E}\left(t\right)n_{S}\left(t\right)+k_{d}\int_{-\infty }^{0}f\left(s\right)n_{E}\left(t+s\right)n_{S}\left(t+s\right)ds,\\ \frac{dn_{P}\left(t\right)}{dt} & =k_{d}\int_{-\infty }^{0}f\left(s\right)n_{E}\left(t+s\right)n_{S}\left(t+s\right)ds, \end{array}$$

where f(s) is density function of delay distribution. In the manuscript [15], theoretical knowledge about kinetics of single molecule associated with the Michaelis–Menten model has been given.

This study was motivated by the desire to describe all possible complexes created during enzyme–substrate interaction with the help of the delay density function. Moreover, we offer an effective method for its parameter estimation in one special case.

## 2. Materials and Methods

In this study, the generalized model of enzyme kinetics describing multi-substrate multi-inhibitor reactions was developed and analyzed. The model is described with the help of differential equations with distributed time delays. This model was applied to investigate the chemical kinetics in the cases of enzyme–substrate and enzyme–substrate–inhibitor interactions.

### 2.1. Generalization to the Multidimensional Case of Competitive Inhibition

In the case of multi-substrate mutli-inhibitor reactions (which are of mixed inhibition type, since they combine the effects of competitive and uncompetitive inhibitions), we consider *M* substrates S_1_, ⋯, S_*M*_, and *N* inhibitors I_1_, ⋯, I_*N*_, acting due to the “mixed” model. Namely, we assume that the substrates and inhibitors are acting independently due to the flowchart of the enzymatic network offered in the work [4], which we call hereinafter multi-substrate mutli-inhibitor enzymatic reactions (MSMIERs).

**Figure 1:**
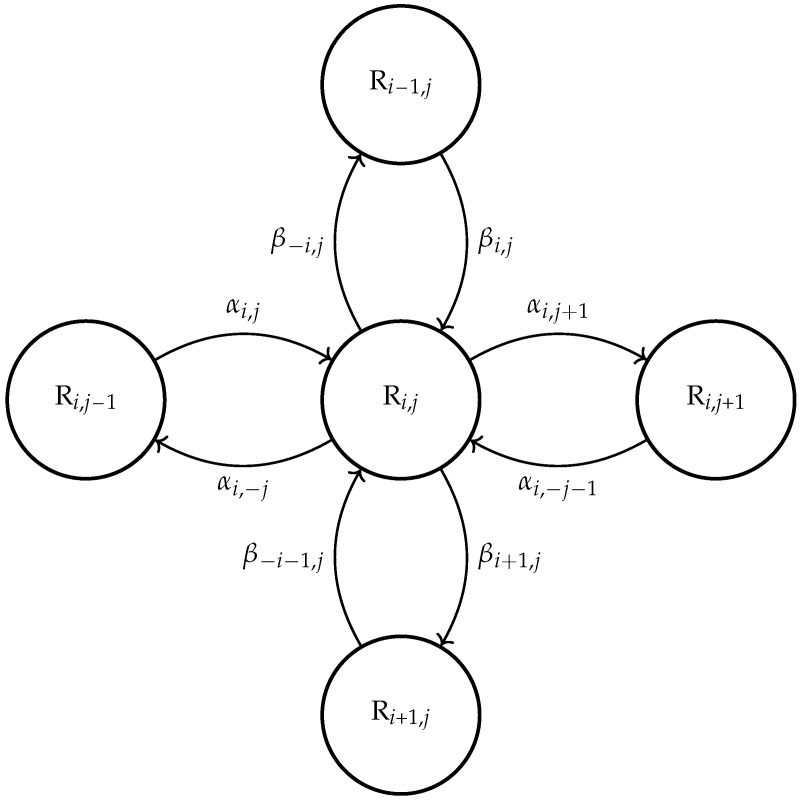
Flowchart of enzymatic reactions with the respect to EIS-complex R_*i,j*_.

In order to generalize the typical Michaelis–Menten model for the case of “multivariate competitive inhibition”, we consider for some *i*, *j* the enzyme–substrate–inhibitor (EIS) complex R_*i,j*_ meaning the binding of the enzyme with the substrates S_1_, ⋯, S_*j*_, and inhibitors I_1_, ⋯, I_*i*_. Enzymatic reactions including R_*i,j*_ are presented in Figure 1 and can be described by the following kinetic reactions:

$$\begin{array}{c} R_{i,j-1}+S_{j}\overset{\alpha_{i,j}}{\underset{\alpha_{i,-j}}{⇌}}R_{i,j}\overset{\gamma_{i,j}}{⟶}E+P_{i,j} \end{array},$$

$$\begin{array}{c} R_{i-1,j}+I_{i}\overset{\beta_{i}}{\underset{\beta_{-i}}{⇌}}R_{i,j} \end{array},$$

$$\begin{array}{c} Ri,j+S_{j+1}\overset{\alpha_{i,j+1}}{\underset{\alpha_{i,-j-1}}{⇌}}R_{i,j+1}\overset{\gamma_{i,j+1}}{⟶}E+P_{i,j+1} \end{array},$$

$$\begin{array}{c} Ri,j+I_{i+1}\overset{\beta_{i+1,j}}{\underset{\beta_{-i-1,j}}{⇌}}R_{i+1,j} \end{array}$$

where P_*i,j*_ is the corresponding reaction products.

Hence, we obtain the system of lattice ordinary differential equations:

(6) $$\begin{array}{cc} \frac{dn_{S_{j}}}{dt}= & \sum_{i=1}^{N}\left\{\alpha_{i,-j}n_{R_{i,j}}-\alpha_{i,j}n_{S_{j}}n_{R_{i,j-1}}\right\},\\ \frac{dn_{I_{i}}}{dt}= & \sum_{j=1}^{M}\left\{\beta_{-i,j}n_{R_{i,j}}-\beta_{i,j}n_{I_{i}}n_{R_{i-1,j}}\right\},\\ \frac{dn_{R_{i,j}}}{dt}= & \alpha_{i,j}n_{S_{j}}n_{R_{i,j-1}}-\left(\gamma_{i,j}+\alpha_{i,-j}\right)n_{R_{i},j}+\beta_{i,j}n_{I_{i}}n_{R_{i-1,j}}-\beta_{i,j}n_{R_{i},j}\\ \; & -\left(\alpha_{i,j+1}n_{S_{j+1}}+\beta_{i+1,j}n_{I_{i+1}}\right)n_{R_{i,j}}+\beta_{-i-1,j}R_{i+1,j}+\alpha_{i,-j-1}R_{i,j+1},\\ \frac{dn_{P_{i,j}}}{dt}= & \gamma_{i,j}n_{R_{i,j}}\\ \; & i=\bar{1,N},\;j=\bar{1,M}. \end{array}$$

Following the ideas of the Brown’s model [10] and introducing time delays needed for binding enzyme E with the substrate S_*j*_ through the EIS complex R_*i,j*_ as $\tau_{i,j}$ and needed for binding E with the inhibitor I_*i*_ through the complex R_*i,j*_ as $h_{i,j}$, we obtain the following system of lattice differential equations with discrete delays:

(7) $$\begin{array}{cc} \frac{dn_{P_{i,j}}}{dt}= & \alpha_{i,j,d}n_{E}\left(t-\tau_{i,j}\right)n_{S_{j}}\left(t-\tau_{i,j}\right),\\ \frac{dn_{S_{j}}}{dt}= & -\sum_{i=1}^{N}\alpha_{i,j,d}n_{S_{j}}\left(t\right)n_{E}\left(t\right),\\ \frac{dn_{E}}{dt}= & \sum_{j=1}^{M}\left(\sum_{i=1}^{N}\alpha_{i,j,d}n_{E}\left(t-\tau_{i,j}\right)n_{S_{j}}\left(t-\tau_{i,j}\right)-\sum_{i=1}^{N}\alpha_{i,j,d}n_{S_{j}}\left(t\right)n_{E}\left(t\right)\right)\\ \; & +\sum_{i=1}^{N}\left(\sum_{j=1}^{M}\beta_{i,j,d}n_{E}\left(t-h_{i,j}\right)n_{I_{i}}\left(t-h_{i,j}\right)-\sum_{j=1}^{M}\beta_{i,j,d}n_{I_{i}}\left(t\right)n_{E}\left(t\right)\right),\\ \frac{dn_{I_{i}}}{dt}= & -\sum_{j=1}^{M}\beta_{i,j,d}n_{I_{i}}\left(t\right)n_{E}\left(t\right), \end{array}$$

Introducing density functions $f_{i,j}$, $g_{i,j}$ for distributed delays, corresponding to discrete delays $\tau_{i,j}$, $h_{i,j}$, $i=\bar{1,N}$, $j=\bar{1,M}$, respectively, we come to the following generalized model based on the system of lattice differential equations with distributed delays:

(8) $$\begin{array}{cc} \frac{dn_{P_{i,j}}}{dt}= & \alpha_{i,j,d}\int_{-\infty }^{0}f_{i,j}\left(s\right)n_{E}\left(t+s\right)n_{S_{j}}\left(t+s\right)ds,\\ \frac{dn_{S_{j}}}{dt}= & -\sum_{i=1}^{N}\alpha_{i,j,d}n_{S_{j}}\left(t\right)n_{E}\left(t\right),\\ \frac{dn_{E}}{dt}= & \sum_{j=1}^{M}\left(\sum_{i=1}^{N}\alpha_{i,j,d}\int_{-\infty }^{0}f_{i,j}\left(s\right)n_{E}\left(t+s\right)n_{S_{j}}\left(t+s\right)ds-\sum_{i=1}^{N}\alpha_{i,j,d}n_{S_{j}}\left(t\right)n_{E}\left(t\right)\right)\\ \; & +\sum_{i=1}^{N}(\sum_{j=1}^{M}\beta_{i,j,d}\int_{-\infty }^{0}g_{i,j}\left(s\right)n_{E}\left(t+s\right)n_{I_{i}}\left(t+s\right)ds-\sum_{j=1}^{M}\beta_{i,j,d}n_{I_{i}}\left(t\right)n_{E}\left(t\right)),\\ \frac{dn_{I_{i}}}{dt}= & -\sum_{j=1}^{M}\beta_{i,j,d}n_{I_{i}}\left(t\right)n_{E}\left(t\right), \end{array}$$

The models (23) and (24) belong to the classes of lattice differential equations with delays. The solution of the model (23) requires the setting of initial conditions on $\left[-\tau_{M},0\right]$, where $\tau_{M}=\max_{i=\bar{1,N},j=\bar{1,M}}\left\{\tau_{i,j},h_{i,j}\right\}$. On the other hand, in order to solve system (24) with the distributed delays, we need initial values for infinite temporal interval. Furthermore, we will present how to reduce it to the finite one.

The dynamics of such type systems in a general case is studied in [16]. Some of our previous results were also related to their behavior [17,18,19]. In the next section, we will consider one simple case allowing us to develop the method of parameter identification for the model such as (24).

## 3. Results

As a result of the general model of enzyme kinetics with distributed delays developed in Section 2.1, we have developed and analyzed models of the enzyme–substrate and enzyme–substrate–inhibitor complexes numerically and experimentally.

### 3.1. Enzyme Kinetics for the Case of One Substrate

In the given section, we apply continuously distributed delays for modeling the enzyme–substrate binding. Pursuing the goal of mapping the processes of combining as well as possible, here we have chosen to use the gamma distribution of delays τ≥0 as:

(9) $$f\left(a,m,\tau_{\min},s\right):=\left\{\begin{array}{cc} 0 & s\leq \tau_{\min},\\ \frac{a^{m+1}}{\Gamma (m+1)}{\left(s-\tau_{\min}\right)}^{m}e^{-a(s-\tau_{\min})} & s>\tau_{\min}, \end{array}\right.$$

where $a,m,\tau_{\min}\geq 0$ are the parameters which are determining the corresponding probability density function. Namely, *m* determines the shape of the density curve, whereas *a* stands for its rate parameter, and $\tau_{\min}\geq 0$ is the minimal possible value of the delay. The distribution was considered earlier in a different context, for example, describing cell maturation times (See [20], pp. 240–243), where an efficient method of distribution parameter estimation based on experimental population data was offered. In addition, the non-symmetricity of gamma distribution fits the processes of chemical kinetics better as compared with the symmetric normal distribution.

Let $\tau_{M}$ be the largest value of the delay considered in (5), which is probably achievable. Assuming τ is a random variable, which is gamma distributed given by *f*, we can estimate its confidence interval with confidence level c∈(0,1) with the help of applying Chebyshev’s inequality to a gamma distribution, resulting in determining the largest value of τ as:

(10) $$\tau_{M}:=E\left(\tau \right)+\sqrt{\frac{\mathrm{Var}(\tau )}{1-c}}=\tau_{m}+\frac{m+1}{a}+\sqrt{\frac{\left(m+1\right)}{a^{2}\left(1-c\right)}}$$

Finally, assuming that binding a substrate to active or regulatory sites requires certain random time $\tau \leq \tau_{M}$, we reformulate the model in the following manner:

(11) $$\begin{array}{cc} \frac{dn_{S}\left(t\right)}{dt} & =-k_{d}n_{E}\left(t\right)n_{S}\left(t\right),\\ \frac{dn_{E}\left(t\right)}{dt} & =-k_{d}n_{E}\left(t\right)n_{S}\left(t\right)+k_{d}\int_{-\tau_{M}}^{0}f\left(s\right)n_{E}\left(t+s\right)n_{S}\left(t+s\right)ds,\\ \frac{dn_{P}\left(t\right)}{dt} & =k_{d}\int_{-\tau_{M}}^{0}f\left(s\right)n_{E}\left(t+s\right)n_{S}\left(t+s\right)ds, \end{array}$$

The dynamics of (11) are determined by probability rate $k_{d}$ and the function of delay density f(s).

### 3.2. Parameter Estimation

Suppose the following initial concentrations are given:

(12) $$n_{S}\left(t\right)\equiv 0,t\in \left[-\tau_{M},0\right),\;n_{S}\left(0\right)=n_{S}^{0},\;n_{E}\left(t\right)\equiv n_{E}^{0},t\in \left[-\tau_{M},0\right]$$

Let $n_{P}\left(t\right)\equiv 0,t\in \left[-\tau_{M},0\right]$.

The idea is to use the model (11) and (12) for estimating the time series of the product with the respect to the enzyme–substrate reaction. In turn, these values correspond to the initial conditions of $n_{S}$, $n_{E}$, and $n_{P}$, which are known from experimental data in advance. Estimation should include the rate constant and the gamma distribution parameters within *f* function in the system given by (11).

In order to adjust the predicted data (product concentration $n_{P}$), we need to make them compatible with the data accessible from the experiment (“expected data”), namely, specific conductance κ, which is related with the molar conductivity $\Lambda_{m}$ as follows:

(13) $$\Lambda_{m}=\frac{\kappa }{n_{P}}.$$

When analyzing the conductivity characteristics of the considered solution, we may conclude that it is a strong electrolyte. It follows from the pH value of BSA (4.5–4.8) [21] that was primarily added when obtaining the CLEA. Hence, we can use Kohlrausch’s law for a solution of a strong electrolyte:

(14) $$\Lambda_{m}=\Lambda_{m}^{0}-K\sqrt{n_{P}}.$$

Combining (13) and (14), we introduce the denotation for the specific conductance obtained from (11), (12) as follows:

(15) $$\kappa_{pred}\left(t\right):=\Lambda_{m}^{0}n_{P,pred}\left(t\right)-K{\left(n_{P,pred}\left(t\right)\right)}^{3/2},$$

where the parameters $\Lambda_{m}^{0}$ and *K* are estimated.

Hence, the enzyme kinetics model (11) depends on six unknown parameters, namely:

(16) $$\Pi =\left\{\begin{array}{c} k_{d},a,m,\tau_{min},\Lambda_{m}^{0},K \end{array}\right\}\in R_{+}^{6}$$

In principle, this set of parameters can be estimated from a given time series of enzyme–substrate interaction. Pursuing the goal to find parameter estimates as accurately as we can, we conducted the experiments with different amounts of the initial dose of the substrate, namely, $n_{S}\left(0\right)=n_{S,i}^{0}$, $i=\bar{1,l}$. Basing on our experiment design, we obtain *l* sets of *n* pointwise experimental data in the time series, say ${\left\{\kappa_{exp,i}\left(t_{j}\right)\right\}}_{i=\bar{1,l},j=\bar{1,n}}$, with $t_{1},t_{2},\ldots ,t_{n}$ being the times of observations. The identification of parameters can be carried out with the following constrained optimization calculations that are expressed in the following form:

(17) $$\left.\begin{array}{cc} \mathrm{minimize} & J\left(\Pi \right),\;\Pi \in R_{+}^{6}\\ \mathrm{subject}\mathrm{to} & c_{i}\left(\Pi \right)\geq \Theta ,\;i=1,2 \end{array}\right\}$$

Here,

(18) $$J\left(\Pi \right):={\left(\sum_{i=1}^{l}\left(\sum_{j=1}^{n}\left({\left(\kappa_{exp,i}\left(t_{j}\right)-\kappa_{pred,i}\left(t_{j}\right)\right)}^{2}\right)\right)\right)}^{1/2}$$

is the objective function and

(19) $$\begin{array}{cc} g_{1}\left(\Pi \right)= & \Pi -\Pi_{lower}\geq \Theta ,\\ g_{2}\left(\Pi \right)= & \Pi_{upper}-\Pi \geq \Theta , \end{array}$$

are inequality constraints, where $\Theta \in R^{6}$ is a null-vector and $\Pi_{lower}$, $\Pi_{upper}$ are the lower and upper bounds for the parameter values, respectively.

The offered solution of the nonlinear optimization problem (17) is based on the COBYLA Algorithm 1, which linearly approximates objective function and constraints on 6-simplex $C=C(\Pi_{0},\Pi_{1},\ldots ,\Pi_{6})$ and optimizes the simplex on each algorithm iteration. The algorithm transforms problem (17) to the problem without constraints with the help of the following objective function:

(20) $$\Phi \left(\Pi \right):=J\left(\Pi \right)+\xi {\left[\max\left\{-g_{i}\left(\Pi \right),i=1,2\right\}\right]}^{+}.$$

We denote its linear approximation on the simplex *C* as ${\hat{\Phi }}_{C}\left(\Pi \right)$. An implementation of COBYLA to the problem (17) can be reformulated as the Algorithm 1. Here, *stop condition* covers the improvement of objective function, the changes of vertices, and allowed number of iterations.
**Algorithm 1:** COBYLA algorithm implementation to the problem (17).
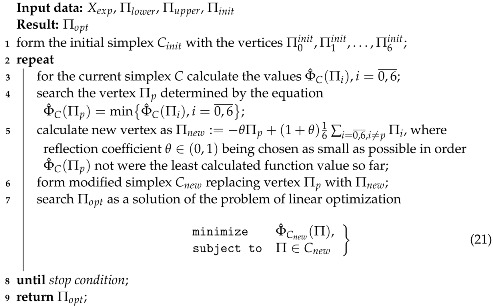


### 3.3. Enzyme Kinetics for Enzyme–Substrate–Inhibitor Interaction

Here, we consider the model (6) in case of M=1, N=1, that is, we have substrate S_1_ and inhibitor I_1_. Based on the multi-substrate multi-inhibitor model, we obtain the system of lattice ordinary differential equations including enzymes E = R_0,0_ and complexes R_0,1_, R_1,0_, and R_1,1_:

(22) $$\begin{array}{cc} \frac{dn_{S_{1}}}{dt}= & \alpha_{0,-1}n_{R_{0,1}}-\alpha_{0,1}n_{S_{1}}n_{R_{0,0}}+\alpha_{1,-1}n_{R_{1,1}}-\alpha_{1,1}n_{S_{1}}n_{R_{1,0}},\\ \frac{dn_{I_{1}}}{dt}= & \beta_{-1,0}n_{R_{1,0}}-\beta_{1,0}n_{I_{1}}n_{R_{0,0}}+\beta_{-1,1}n_{R_{1,1}}-\beta_{1,1}n_{I_{1}}n_{R_{0,1}},\\ \frac{dn_{R_{0,0}}}{dt}= & -\left(\gamma_{0,0}+\alpha_{0,-0}\right)n_{R_{0,0}}-\beta_{0,0}n_{R_{0,0}}\\ \; & -\left(\alpha_{0,1}n_{S_{1}}+\beta_{1,0}n_{I_{1}}\right)n_{R_{0,0}}+\beta_{-1,0}n_{R_{1,0}}+\alpha_{0,-1}n_{R_{0,1}},\\ \frac{dn_{R_{1,0}}}{dt}= & -\left(\gamma_{1,0}+\alpha_{1,-0}\right)n_{R_{1,0}}+\beta_{1,0}n_{I_{1}}n_{R_{0,0}}-\beta_{1,0}n_{R_{1,0}}\\ \; & -\alpha_{1,1}n_{S_{1}}n_{R_{1,0}}+\alpha_{1,-1}n_{R_{1,1}},\\ \frac{dn_{R_{0,1}}}{dt}= & \alpha_{0,1}n_{S_{1}}n_{R_{0,0}}-\left(\gamma_{0,1}+\alpha_{0,-1}\right)n_{R_{0,1}}-\beta_{0,1}n_{R_{0,1}}\\ \; & +\beta_{1,1}n_{I_{1}})n_{R_{0,1}}+\beta_{-1,1}n_{R_{1,1}},\\ \frac{dn_{R_{1,1}}}{dt}= & \alpha_{1,1}n_{S_{1}}n_{R_{1,0}}-\left(\gamma_{1,1}+\alpha_{1,-1}\right)n_{R_{1,1}}+\beta_{1,1}n_{I_{1}}n_{R_{0,1}}-\beta_{1,1}n_{R_{1,1}},\\ \frac{dn_{P_{i,j}}}{dt}= & \gamma_{i,j}n_{R_{i,j}}\\ \; & i=\bar{0,1},\;j=\bar{0,1},\;i=j\neq 0. \end{array}$$

Following the ideas of Brown’s model [10], and introducing time delays needed for binding enzyme E with the substrate S_1_ through the ES complex R_0,1_ as $\tau_{0,1}$ through the EIS complex R_1,1_ as $\tau_{1,1}$, for binding E with the inhibitor I_1_ through the EI complex R_1,0_ as $h_{1,0}$, and through the EIS complex R_1,1_ as $h_{1,1}$, we obtain the following system of lattice differential equations with discrete delays:

(23) $$\begin{array}{cc} \frac{dn_{P_{0,1}}}{dt}= & \alpha_{0,1,d}n_{E}\left(t-\tau_{0,1}\right)n_{S_{1}}\left(t-\tau_{0,1}\right),\\ \frac{dn_{P_{1,1}}}{dt}= & \alpha_{1,1,d}n_{E}\left(t-\tau_{1,1}\right)n_{S_{1}}\left(t-\tau_{1,1}\right),\\ \frac{dn_{S_{1}}}{dt}= & -\left(\alpha_{0,1,d}+\alpha_{1,1,d}\right)n_{S_{1}}\left(t\right)n_{E}\left(t\right),\\ \frac{dn_{E}}{dt}= & \alpha_{0,1,d}n_{E}\left(t-\tau_{0,1}\right)n_{S_{1}}\left(t-\tau_{0,1}\right)+\alpha_{1,1,d}n_{E}\left(t-\tau_{1,1}\right)n_{S_{1}}\left(t-\tau_{1,1}\right)\\ \; & -\left(\alpha_{0,1,d}+\alpha_{1,1,d}\right)n_{S_{1}}\left(t\right)n_{E}\left(t\right)\\ \; & +\beta_{1,0,d}n_{I_{1}}\left(t-h_{1,0}\right)n_{E}\left(t-h_{1,0}\right)+\beta_{1,1,d}n_{I_{1}}\left(t-h_{1,1}\right)n_{E}\left(t-h_{1,1}\right)\\ \; & -\left(\beta_{1,0,d}+\beta_{1,1,d}\right)n_{I_{1}}\left(t\right)n_{E}\left(t\right),\\ \frac{dn_{I_{1}}}{dt}= & -\left(\beta_{1,0,d}+\beta_{1,1,d}\right)n_{I_{1}}\left(t\right)n_{E}\left(t\right), \end{array}$$

Introducing density functions $f_{0,1}$, $f_{1,1}$, $g_{1,0}$, and $g_{1,1}$ for distributed delays, corresponding to discrete delays $\tau_{0,1}$, $\tau_{1,1}$, $h_{1,0}$, and $h_{1,1}$ respectively, we come to the following model based on the system of differential equations with distributed delays:

(24) $$\begin{array}{cc} \frac{dn_{P_{0,1}}}{dt}= & \alpha_{0,1,d}\int_{-\tau_{M,0,1}}^{0}f_{0,1}\left(s\right)n_{E}\left(t+s\right)n_{S_{1}}\left(t+s\right)ds,\\ \frac{dn_{P_{1,1}}}{dt}= & \alpha_{1,1,d}\int_{-\tau_{M,1,1}}^{0}f_{1,1}\left(s\right)n_{E}\left(t+s\right)n_{S_{1}}\left(t+s\right)ds,\\ \frac{dn_{S_{1}}}{dt}= & -\left(\alpha_{0,1,d}+\alpha_{1,1,d}\right)n_{S_{1}}\left(t\right)n_{E}\left(t\right),\\ \frac{dn_{E}}{dt}= & \alpha_{0,1,d}\int_{-\tau_{M,0,1}}^{0}f_{0,1}\left(s\right)n_{E}\left(t+s\right)n_{S_{1}}\left(t+s\right)ds\\ + & \alpha_{1,1,d}\int_{-\tau_{M,1,1}}^{0}f_{1,1}\left(s\right)n_{E}\left(t+s\right)n_{S_{1}}\left(t+s\right)ds-\left(\alpha_{0,1,d}+\alpha_{1,1,d}\right)n_{S_{1}}\left(t\right)n_{E}\left(t\right)\\ + & \beta_{1,0,d}\int_{-h_{M,1,0}}^{0}g_{1,0}\left(s\right)n_{I_{1}}\left(t+s\right)n_{E}\left(t+s\right)ds\\ + & \beta_{1,1,d}\int_{-h_{M,1,1}}^{0}g_{1,1}\left(s\right)n_{I_{1}}\left(t+s\right)n_{E}\left(t+s\right)ds-\left(\beta_{1,0,d}+\beta_{1,1,d}\right)n_{I_{1}}\left(t\right)n_{E}\left(t\right),\\ \frac{dn_{I_{1}}}{dt}= & -\left(\beta_{1,0,d}+\beta_{1,1,d}\right)n_{I_{1}}\left(t\right)n_{E}\left(t\right), \end{array}$$

Following (10), we let:

$$\begin{array}{cc} \; & \tau_{M,i,j}:=\tau_{m,i,j}+\frac{m_{i,j}+1}{a_{i,j}}+\sqrt{\frac{\left(m_{i,j}+1\right)}{a_{i,j}^{2}\left(1-c\right)}},\;h_{M,i,j}:=h_{m,i,j}+\frac{k_{i,j}+1}{b_{i,j}}+\sqrt{\frac{\left(k_{i,j}+1\right)}{b_{i,j}^{2}\left(1-c\right)}},\\ \; & i,j\in \{0,1\},\;i=j\neq 0, \end{array}$$

where $a_{i,j}$, $m_{i,j}$, $\tau_{m,i,j}$, and $b_{i,j}$, $k_{i,j}$, $h_{m,i,j}$ are the parameters of the corresponding density functions $f_{i,j}$ and $g_{i,j}$ as in (9) and *c* is the confidence level.

## 4. Experimental Study

### 4.1. Enzyme–Substrate Interaction

Enzyme biosensors consist of enzymes immobilized at the surface of the transducer. Hence, an immobilization step with the help of Bovine Serum Albumin (BSA) is very important as it affects the sensitivity and selectivity of biosensors. Enzyme products may be electroactive, meaning their activity may be followed by amperometry.

In our study, Acetylcholinesterase (AChE) as an enzyme has been used, since it has a very high catalytic activity. AChE is often used to design biosensors based on the inhibition analysis.

Since conductivity (specifically, electrolytic conductivity) is the ability of a substance to conduct an electric current, in solvents where electrical conductivity is present, particularly water, ionisation will provide the necessary carriers. The electrical conductivity of a solution is determined by both the physical characteristics of the carriers and the medium. The measured conductivity comes from the ions of dissolved substances. The preliminary measurement has been conducted in aqueous solutions.

The studies were carried out in aqueous solutions using the following substances: protein—BSA; enzyme—enzyme acetylcholinesterase (AChE); and substrate—Acetylcholine chloride (AChCl). The substances were purchased from Sigma Aldrich.

To the complex formed with BSA of 2 mg/mL (volume of 4 mL) and enzyme of 2 mg/mL (volume of 0.1 mL) (the complex is known as cross-linked enzyme aggregate (CLEA) [22]), substrate of 2 mg/mL in different volumes (0.1 mL, 0.3 mL, 0.9 mL, 1.5 mL) was added. As a result, four samples were obtained:

Sample 1. BSA + enzyme + 0.1 mL of substrate;
Sample 2. BSA + enzyme + 0.3 mL of substrate;
Sample 3. BSA + enzyme + 0.9 mL of substrate;
Sample 4. BSA + enzyme + 1.5 mL of substrate.

The conductivity of the BSA + enzyme + substrate complex was tested by using a specially constructed measuring setup, during 500 s with signal sampling every 5 s. There were 100 conductivity measurements made during 500 s.

#### 4.1.1. Experimental Data

As a result of the experiments, the dependence of the conductivity changes in time was obtained. In the initial stage (0–249 s), the conductivity of the BSA–enzyme complex was tested. When the conductivity remained constant, in the 250th second of the measurement, a substrate of different concentration was added to the BSA–enzyme complex. The addition of a substrate rapidly changed the conductivity of the resulting complex, which may indicate a rapid chemical reaction. By comparing the changes in conductivity as a function of the volume of the substrate added, it can be said that the larger the volume of the substrate added, the greater the change in conductivity was and the more dynamic the increase in conductivity was.

#### 4.1.2. Parameter Estimation for the Experimental Study

Algorithm 1 was implemented in R package. For the integration of (11), Julia calling was used. Initial parameter values together with the parameter bounds are presented in Table 1. The solution of the optimization problem (17) is displayed in the column $\Pi_{opt}$. The root mean squared error of the prediction with the obtained model throughout all data series (i.e., for different initial substrate volumes) is 48.74105. The number of iterations was 50.

**Figure 2:**
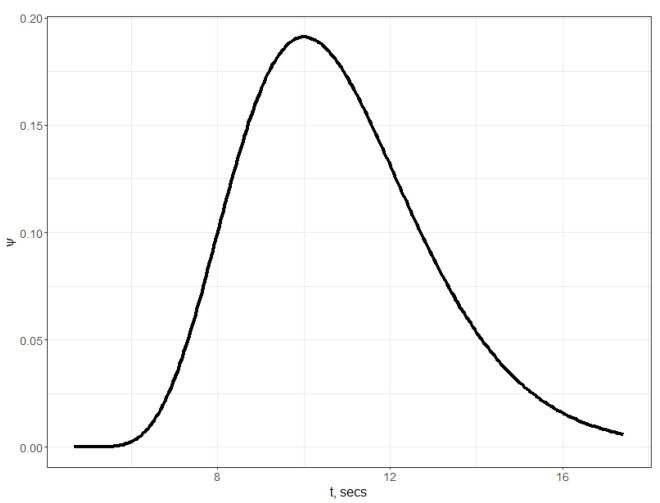
Estimated density of distributed delay f(s) for model (11).

**Figure 3:**
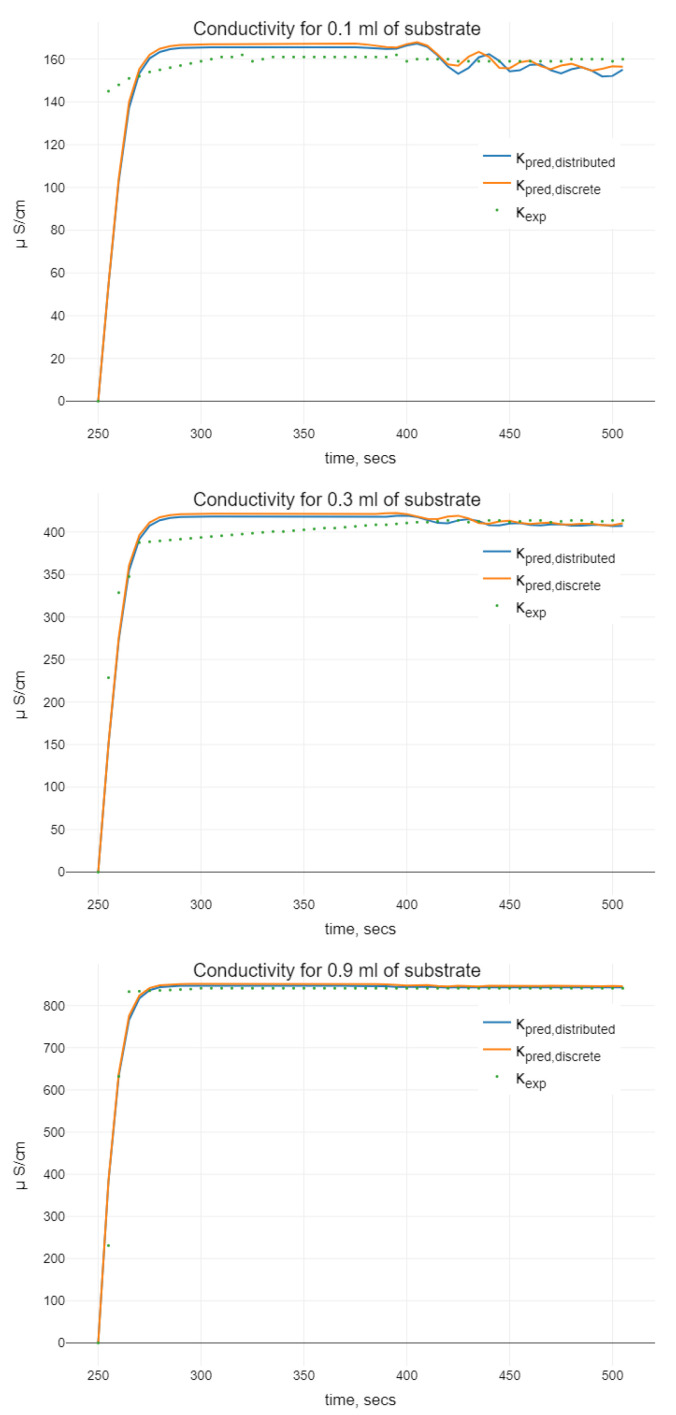

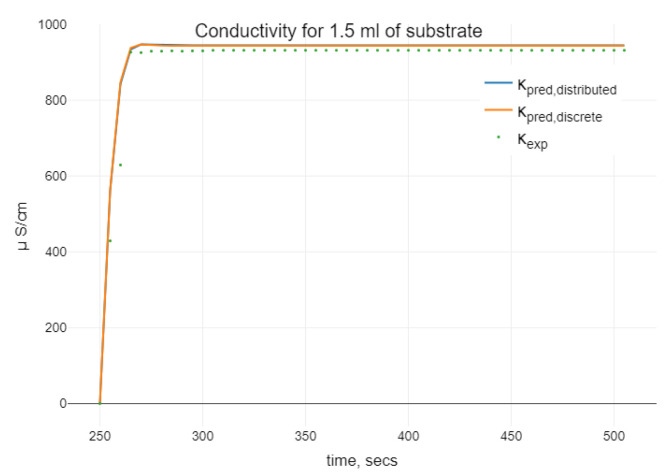
Plots of expected vs predicted trajectories with the help of (11). Additionally, a comparison of modeling with the help of Brown’s model (4) with discrete delay is shown: — $\kappa_{pred,distributed}$, — $\kappa_{pred,discrete}$ · $\kappa_{exp}$.

The density function f(s) for estimated parameters is shown on Figure 2. In Figure 3, the results of integration of (11) and (4) for different initial volumes of substrate which are based on the values of parameters estimated are shown.

### 4.2. Enzyme–Substrate–Inhibitor Interaction

The second experimental study used butyryl cholinesterase (BuChE) (EC 3.1.1.8, from Horse Serum) with a specific activity of 13 U/mg solid bovine albumin (fraction V, 98% purity), butyryl choline chloride (BuChCl) (98% purity), a-chaconine (95% purity) from potato sprouts, and glutaraldehyde (grade II, 25% aqueous solution), which were purchased from Sigma-Aldrich Chemie GmbH (Steinheim, Germany). All other reagents were of analytical grade and were used without any further treatment.

Biologically active membranes were formed by cross-linking butyryl cholinesterase with BSA on the transducer surface in a saturated glutaraldehyde vapour. The mixture containing 5% (*w*/*v*) butyryl cholinesterase, 5% (*w*/*v*) BSA, and 10% (*w*/*v*) glycerol in 20 mM phosphate buffer (pH 7.2) was deposited on the sensitive surface of one transducer by the drop method, while the mixture of 10% (*w*/*v*) BSA and 10 % (*w*/*v*) glycerol in 20 mM phosphate buffer (pH 7.2) was placed on the surface of a reference transducer. The sensor chip was then placed in a saturated glutaraldehyde vapor. After a 30 min exposure in glutaraldehyde, the membranes were dried at room temperature for 15 min.

All measurements were performed in daylight at room temperature in an open glass vessel filled with a vigorously stirred 5 mM phosphate buffer solution, pH 7.2. The 200 mM stock solution of BuChCl in deionised H${}_{2}$O, and 2 mM stock solution of the a-chaconine in 5 mM acetic acid were prepared. The concentrations of substrates and inhibitors were adjusted by adding defined volumes of the stock solution of proper concentration. The differential output signal between the measuring and reference Ion-Sensitive Field Effect Transistors (ISFETs) was registered using portable device. After the response measurement (determination of enzyme inhibition), the initial enzyme activity was restored by washing out the biosensor enzymatic membrane in the working buffer solution for 10 to 15 min.

### 4.3. Numerical Simulation of Enzyme–Substrate–Inhibitor Model

**Figure 4:**
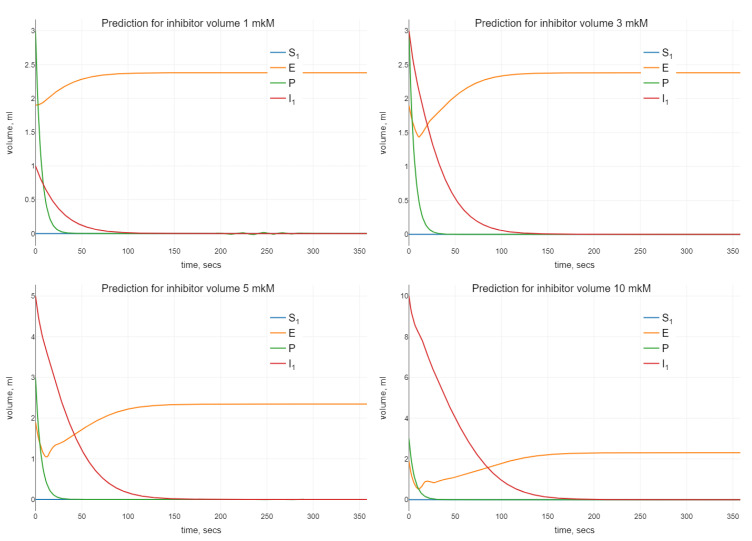
Numerical simulation with the help of (24) for different initial values of inhibitors.

Algorithm 1 was modified for the estimation of the parameters of the model (24). The optimal values of the parameters were used for the simulation using different initial values for the inhibitor. The visualization of the simulations results is presented in Figure 4.

## 5. Discussion and Conclusions

**Figure 5:**
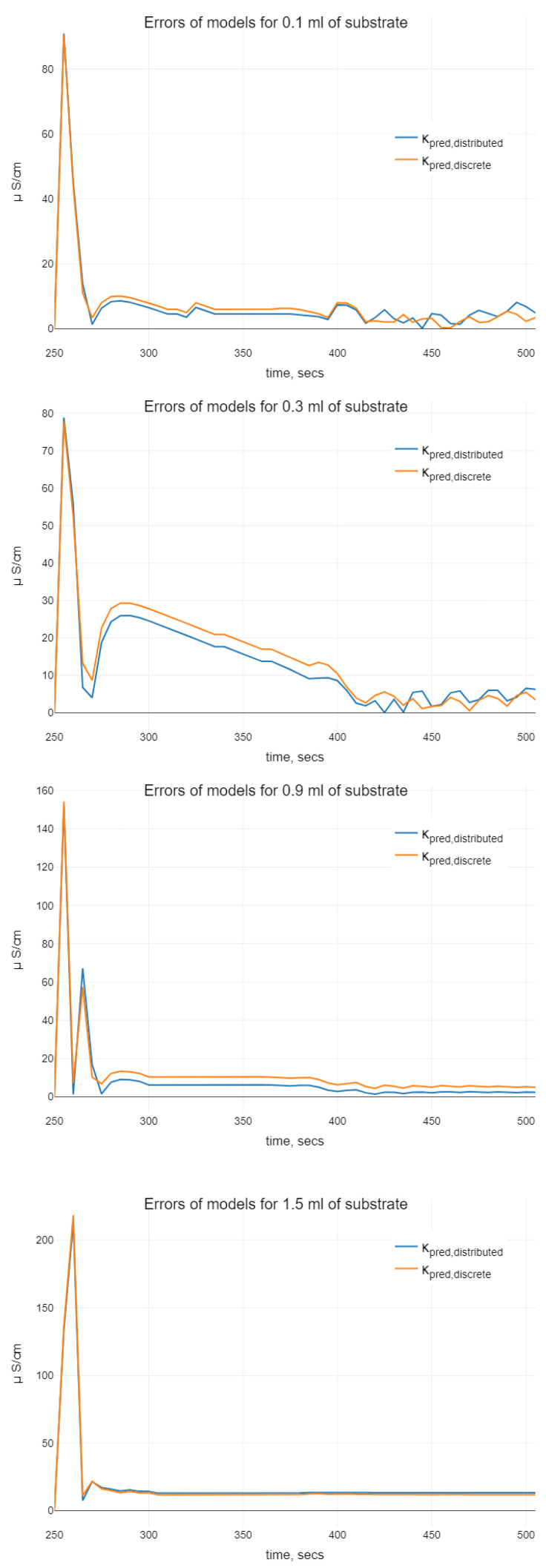
Error analysis of predicted trajectories with the help of (11) and (4): — denotes the errors of $\kappa_{pred,distributed}$, — denotes the errors of $\kappa_{pred,discrete}$.

While analyzing the models of (11) and (4), with the help of plots on Figure 3 and Figure 5, we can conclude the following. It is clearly seen that “undesirable” oscillations in the trajectories are appearing at the smaller initial volumes of the substrate. Then, oscillations disappear for bigger ones. There are some differences between trajectories of models with distributed and discrete delay (we mean that the model with discrete delay (4) is constructed for the mean value of delay τ obtained using the density function f(s), i.e., for $\tau =\tau_{m}+\frac{m+1}{a}$τ) observable on the plots. Namely, we see differences in the phases of oscillations. Using the distributed delay, the oscillation starts later. Moreover, for the bigger values of initial substrate volume, the oscillations for the distributed delay model disappear (as it can be seen in case of 1.5 mL of substrate). The amplitudes of the oscillations at the smaller values $n_{S}^{0}$ are similar, whereas by increasing the initial substrate amount, the oscillations for distributed delay models are not seen. Hence, when comparing two kinds of delay models, which both demonstrate oscillations in certain sense, we conclude that (11) is more chemically adequate for bigger values of the initial substrate dose than the discrete delay model.

When analyzing the enzyme–substrate–inhibitor model (24), we observe a similar oscillating behavior when increasing the initial amount of inhibitor. Namely, as it can be seen in Figure 4, small oscillations in *P* disappear when we increase $n_{I}^{0}$.

In summary, we can conclude that the modeling of the enzyme kinetics based on dynamic systems with distributed delays can make the usage of the delayed differential equations more attractive since it shows more adequate chemical behavior, reducing oscillations when compared with the discrete delays. This is due to more continuous right-sides of the differential equations with distributed delays in comparison to discrete ones. On the other hand, such types of models are complicated enough to demonstrate complex nonlinear behavior, e.g., with the help of using parameters of the density of the delay distribution.

One should bear in mind the computational complexity of the respective models. So, in the case of multi-substrate multi-inhibitor modeling using the model (6), we need to solve the system of N+M+2NM equations. Using the models with delay (23) or (24), the number of equations determined by the number of phase coordinates is N+M+NM+1, which is NM−1 smaller. In the case of complicated computing, such as multi-iteration process optimization for the purpose of parameter estimation, this difference in the order of computational complexity is crucial. Even in the case of Julia computing, numerical integration of the systems of differential equations is time-consuming when the system order or accuracy is growing.

Algorithm 1 can be extended to the parameter estimation of *M*-substrate *N*-inhibitor model (24). In such a case, the parameters to be estimated are reaction rates $\alpha_{i,j,d}$, $\beta_{i,j,d}$, three parameters for each density $f_{i,j}\left(s\right)$ and $g_{i,j}\left(s\right)$, $i=\bar{1,N}$, $j=\bar{1,M}$, $\Lambda_{m}^{0}$, and *K*. That is, we obtain 5NM+2 parameters at the whole, which leads to the corresponding computational complexity. The convergence of such an algorithm is also essential, which is closely dealt with in the search of the initial approximation. We think that in some special cases, the approach presented in Section 3.2 could be applied for experimental data as well.

The usage of delay models for enzyme kinetics is of importance in the multi-substrate multi-inhibitor cases, as they demonstrate more complex qualitative behavior than the models without delay; for example, outbreaks can repeat in the delayed model, whereas we do not observe this in the case without delay. In the future, we will try to evidence this using experimental data.

Here, we only mention that the application of the delayed models is also preferable from the viewpoint of availability of experimental data. Namely, a typical Michaelis–Menten model is based on the formation of multiple complexes. Their concentrations cannot be assessed from a research point of view. In fact, we measure the product of the reaction through the conductivity of the solution. By using time delays, we do not consider concentrations, but we consider the appearance and disappearance of complexes without having to give concentrations. Moreover, delays can be identified with the help of the algorithm offered above.

In the given work, the study of an enzyme kinetics is based on the chemical response using conductivity. The problem is that in modeling, with the help of mass action law, the exact value of the concentration of the substance is required, whereas the experimental results offer the conductivity. As a rule, they use concentration–conductivity dependencies as linear ones, although it has been shown by Kohlrausch’s studies that the relationships are more complex. In this paper, the assumption for strong electrolytes is used, allowing us to apply the corresponding modification of Kohlrausch law, for which parameters $\Lambda_{m}^{0}$ and *K* were experimental with the help of Algorithm 1. In the future, on the basis of the scheme presented in the paper concerning chemical reactions, we plan to carry out experiments that would take into account conductivity tests depending on many substances, applied one after another and also applied simultaneously. Therefore, the effect of time delays in multi-substrate multi-inhibitor enzyme kinetics modeling is still an open problem, requiring the development of many techniques to apply real experimental data, which offers further possibilities to explore it.

## Figures and Tables

**Table 1:** Initial values and bounds for parameter identification based on the Algorithm 1. Column $\Pi_{opt}$ contains the solution of (17).

	$\Pi_{\mathrm{init}}$	$\Pi_{\mathrm{lower}}$	$\Pi_{\mathrm{upper}}$	$\Pi_{\mathrm{opt}}$
$k_{d}$	0.04	$1\times 10^{-10}$	1	0.04042714
*a*	1	$1\times 10^{-10}$	1000	1.255818
*m*	20	$1\times 10^{-10}$	1000	6.703709
$\tau_{min}$	5	$1\times 10^{-4}$	1000	4.673685
$\Lambda_{m}^{0}$	6000	$1\times 10^{-10}$	$1\times 10^{6}$	729.2215
*K*	50	$1\times 10^{-10}$	$1\times 10^{6}$	246.2885

## Data Availability

The following files are freely available on https://github.com/marceniuk/R-electrochemical-biosensor (Accessed date is 4 November 2021): (1) BrownDistribute.R: file with the script in R language with Julia calling to implement Algorithm 1 for the enzyme–substrate model; (2) ESIPBrownDistribute.R: file with the script in R language for the enzyme–substrate–inhibitor model; (3) Conductivities.csv: experimental data of conductivity for different initial volumes of substrate; (4) ConductivitiesESIP.csv: experimental data of conductivity for different initial volumes of inhibitor.

## Abbreviations

The following abbreviations are used in this manuscript:

IR1CMM	Irreversible one-complex Michaelis-Menthen
IR1CB	Irreversible one-complex Brown’s
MSMIER	Multi-substrate mutli-inhibitor enzymatic reaction
ESI	Enzyme-substrate-inhibitor
